# NnU-Net versus mesh growing algorithm as a tool for the robust and timely segmentation of neurosurgical 3D images in contrast-enhanced T1 MRI scans

**DOI:** 10.1007/s00701-024-05973-8

**Published:** 2024-02-20

**Authors:** Mathijs de Boer, Tessa M. Kos, Tim Fick, Jesse A. M. van Doormaal, Elisa Colombo, Hugo J. Kuijf, Pierre A. J. T. Robe, Luca P. Regli, Lambertus W. Bartels, Tristan P. C. van Doormaal

**Affiliations:** 1 Image Sciences Institute, University Medical Center Utrecht, Heidelberglaan 100, 3584 CX Utrecht, The Netherlands; 2 Department of Neuro-Oncology, Princess Máxima Center for Pediatric Oncology, Utrecht, The Netherlands; 3 Department of Neurosurgery, University Medical Center Utrecht, Utrecht, The Netherlands; 4 Department of Neurosurgery, University Hospital of Zürich, Zurich, Switzerland

**Keywords:** Artificial intelligence, Augmented reality, Deep learning, Neurosurgical planning, Segmentation, Visualization

## Abstract

**Purpose:**

This study evaluates the nnU-Net for segmenting brain, skin, tumors, and ventricles in contrast-enhanced T1 (T1CE) images, benchmarking it against an established mesh growing algorithm (MGA).

**Methods:**

We used 67 retrospectively collected annotated single-center T1CE brain scans for training models for brain, skin, tumor, and ventricle segmentation. An additional 32 scans from two centers were used test performance compared to that of the MGA. The performance was measured using the Dice-Sørensen coefficient (DSC), intersection over union (IoU), 95th percentile Hausdorff distance (HD95), and average symmetric surface distance (ASSD) metrics, with time to segment also compared.

**Results:**

The nnU-Net models significantly outperformed the MGA (*p* < 0.0125) with a median brain segmentation DSC of 0.971 [95CI: 0.945–0.979], skin: 0.997 [95CI: 0.984–0.999], tumor: 0.926 [95CI: 0.508–0.968], and ventricles: 0.910 [95CI: 0.812–0.968]. Compared to the MGA’s median DSC for brain: 0.936 [95CI: 0.890, 0.958], skin: 0.991 [95CI: 0.964, 0.996], tumor: 0.723 [95CI: 0.000–0.926], and ventricles: 0.856 [95CI: 0.216–0.916]. NnU-Net performance between centers did not significantly differ except for the skin segmentations Additionally, the nnU-Net models were faster (mean: 1139 s [95CI: 685.0–1616]) than the MGA (mean: 2851 s [95CI: 1482–6246]).

**Conclusions:**

The nnU-Net is a fast, reliable tool for creating automatic deep learning-based segmentation pipelines, reducing the need for extensive manual tuning and iteration. The models are able to achieve this performance despite a modestly sized training set. The ability to create high-quality segmentations in a short timespan can prove invaluable in neurosurgical settings.

**Supplementary Information:**

The online version contains supplementary material available at 10.1007/s00701-024-05973-8.

## Introduction

Three-dimensional (3D) visualization is increasingly being recognized as a crucial tool in neurosurgical interventions [25], often in combination with mixed reality (MxR) glasses and/or microscopic overlays of meshes [3, 6, 12, 18, 19, 28]. These advances enhance a surgeon’s ability to plan and execute complex procedures. A key prerequisite for such visualization is the creation of high quality segmentations, which can be done manually, or (semi-)automatically. The process of generating high-quality manual segmentations is a time-consuming process and requires significant training. Consequently, automated generation of segmentations has emerged as an active and pressing[10, 24, 26, 33].

The use of deep learning (DL) in semantic segmentation of medical images has seen a significant rise. Early deep neural networks (DNN) were generally based on AlexNet [17], VGG [32], and ResNet [9], which were modified to perform voxel-wise classification. These models would often require thousands of training samples to perform well, which made them less suitable for the limited datasets available for clinical applications. Additionally, due to the pixel-wise nature of these models, creating predictions on 3D voxel volumes would result in a very inefficient process.

The introduction of the U-Net [30] presented a significant step forward. Originally designed to efficiently segment cells in histopathological slides, the U-Net is particularly strong at training on smaller datasets and is able to classify several voxels in one pass [30], enhancing performance by better considering the relationships between surrounding structures. Since its introduction, the U-Net has seen a wide adoption with many variations and applications [5, 31].

A notable development was the nnU-Net [13], which included dataset-specific features to guide preprocessing and architectural choices. These features are extracted from a dataset automatically, making it possible to fully automatically generate a new pipeline with little to no human intervention [13]. The creators, Isensee et al., argue that this emphasis on the extraction of dataset-specific features is more effective than changing the U-Net’s core architecture, hence the name “no new U-Net.”

A mathematical mesh-growing algorithm (MGA) was developed in an effort to provide surgeons easier access to automated segmentation tools for neurosurgical planning. This method was validated for segmentation of contrast enhancing tumors and ventricles [7, 35], Despite its effectiveness, the MGA has to be hand-tailored to specific anatomical structures and may still require manual fine-tuning for a perfect match [7].

The growing integration and exploration of MxR tools in neurosurgical practices indicates a future where accurate, on-demand segmentations are required. Whether used for optimizing a surgical planning or refining surgical training through virtual rehearsals, high-quality segmentations are vital to the further adoption of these technologies. Additionally, by introducing nnU-Net to a neurosurgical audience, we aim to allow future research to circumvent the effort required to create a custom segmentation pipeline. This will allow them to focus on exploring larger clinical questions and impacts, achieving more meaningful medical advancements.

The presented study aims to demonstrate nnU-Net as an efficient tool for creating an automatic pipeline for brain, skin, ventricles, and tumor segmentation in T1-weighted contrast-enhanced (T1CE) magnetic resonance imaging (MRI) scans. We benchmark its performance against the previously developed, non-DL, mathematical MGA, providing a comparative view of these two different approaches to segmentation.

## Methods

### Data

The data was sourced from two leading academic neurosurgical hospitals (center A and center B). The training set consisted of 67 T1CE scans, exclusively from center A, each corresponding to a distinct patient with one or more contrast-enhancing tumors. Center A predominantly uses Philips Ingenia and Achieva scanners. Center B uses a wide range of scanner manufacturers and models through referring centers. The training data was originally collected and segmented for use in two previous studies [7, 35], which explored automatic tumor and ventricle segmentations. Testing data, consisting of a random selection of contrast enhancing lesions of minimally 1 cm, was collected separately from the training samples in both centers (*n* = 15 for center A and *n* = 17 for center B). These scans were excluded from model training or any other algorithmic development and were used as a reference standard.

The data collected by Fick et al. [7] consisted of 50 T1CE scans of patients with at least one contrast-enhancing tumor with a volume of no less than 5 cc, and a scan volume of no less than 100 slices at center A. The data collected by van Doormaal et al. [35] consisted of 46 scans, from both centers A and B, of patients who were admitted for intracranial surgery. No further inclusion criteria were used. Data from center B was not included in our training set. The scans from center A were made between August 2018 and November 2020.

### Manual segmentation

Not all patient scans in the training (*n* = 67) and test (*n* = 32) sets were fully segmented. The choice to segment or not to segment a certain anatomy in a patient was based on balancing the amount of effort required versus the potential performance increase an additional segmentation would provide. Mainly, smaller and more variable structures require more segmentations, while larger and less variable ones do not. Each segmentation was checked for quality by an experienced neurosurgeon. For an overview of available segmentations in our train and test sets, refer to Table 1.

**Table 1:** The number of available (semi-)manual segmentations

Anatomy	Train (*n* = 67)	Test (*n* = 32)
Brain	30	23
Skin	60	32
Ventricles	60	32
Tumor	61	30

Initial brain segmentations were created using CAT12 v12.8.1 for patients with an available T1 non-contrast scan of suitable quality. These segmentations were manually refined in 3D Slicer v5.4.0 to eliminate any artefacts caused by patient motion or the presence of metal objects.. The resulting segmentations were then used to bootstrap a MONAI Label v0.8.0 active learning session, in which a small supporting neural network is used to provide best-guess initial segmentations which are then manually checked and corrected where required before inclusion in the dataset.

### NnU-Net model development and training

The nnU-Net codifies best-practice pre-processing and architectural considerations automatically based on features of the provided dataset. These features include modality, spacing, and scan sizes, which can be extracted from the dataset automatically. Based on these features, a number of decisions are made to adjust U-Net architecture and data processing steps. With this method, hyperparameters are selected based on a-priori domain knowledge, resulting in a more robust model. For further technical information, we refer to the original paper by Isensee et al. [13].

We trained four separate models for each anatomical structure using the training data and the nnU-Net v2.2.1 tools. Standard settings were used for each training run. A model was trained for 1000 epochs, 250 steps per epoch, two random patches per step, using a DSC with Cross Entropy loss. NnU-Net is designed around the lack of testing data, using a fivefold cross-validation set up by default. As we were in possession of a separate testing set, we disabled this functionality, and trained on all data instead. Each anatomical structure was assigned its own dataset, and was preprocessed independently. To mitigate false positives in our brain segmentations, we employed a straightforward post-processing step that retained only the largest distinct island in the predicted segmentations. This step capitalized on the a priori knowledge that the brain is one large, continuous structure.

### Mesh-growing algorithm

In the MGA, the scans are pre-processed to a mathematical format. The computational system utilizes image data and a-priori anatomical information to determine initial anatomical structure boundaries. The intermediate results are used to guide the segmentation of the of the tissues of interest with region-growing and watershed algorithms. An iterative process optimizes the segmentations further and includes any regions that were missed in the initial steps. The original application of the MGA was the segmentation of orbital volumes [14, 27], which was later expanded to wrist [34], ankle [16], and intracranial anatomies [7, 35]. This approach is fully deterministic, using classic numerical algorithms and is not based on any DL techniques.

### Experiments

To evaluate the performance of the trained nnU-Net models, we generated predictions on the test scans. The resulting outputs were compared with the available manual segmentations. We used the Dice-Sørensen coefficient (DSC), intersection over union (IoU), 95th percentile Hausdorff distance (HD95), and average symmetric surface distance (ASSD) for numerical performance evaluation. The implementations of these metrics were provided by MONAI v1.3.0, paired with PyTorch v2.0.1. All metrics were collected on a per-patient basis, and did not include the background class. The choice of these particular metrics was intentional to ensure that the weaknesses of any individual metric were mitigated by incorporation of the others [21, 29].

We also compared the time required to create the automatic segmentations. For nnU-Net, we use the automatically provided timing with all predictions. Given that the MGA was ran in a cloud environment that only provides timing to the nearest full minute and since the MGA delivers all predictions at once, we added the nnU-Net times per patient to simulate a sequential segmentation setup.

### Statistical analyses

A Shapiro–Wilk test showed that the resulting scores were not normally distributed, leading us to use a Mann–Whitney *U* test to identify the existence of any statistically significant performance difference between (1) the two methods and (2) between centers A and B for the nnU-Net segmentations. All statistical tests were implemented by SciPy v1.11.4.

The alternative hypotheses available in the used Mann–Whitney *U* test implementation were utilized to assess whether our models had a statistically better performance than the MGA. We assessed whether the nnU-Net models scored significantly higher for the DSC and IoU scores, and significantly lower for the HD95 and ASSD scores. We calculated our Bonferroni adjusted *p*-value to be 0.0125.

## Results

**Fig. 1:**
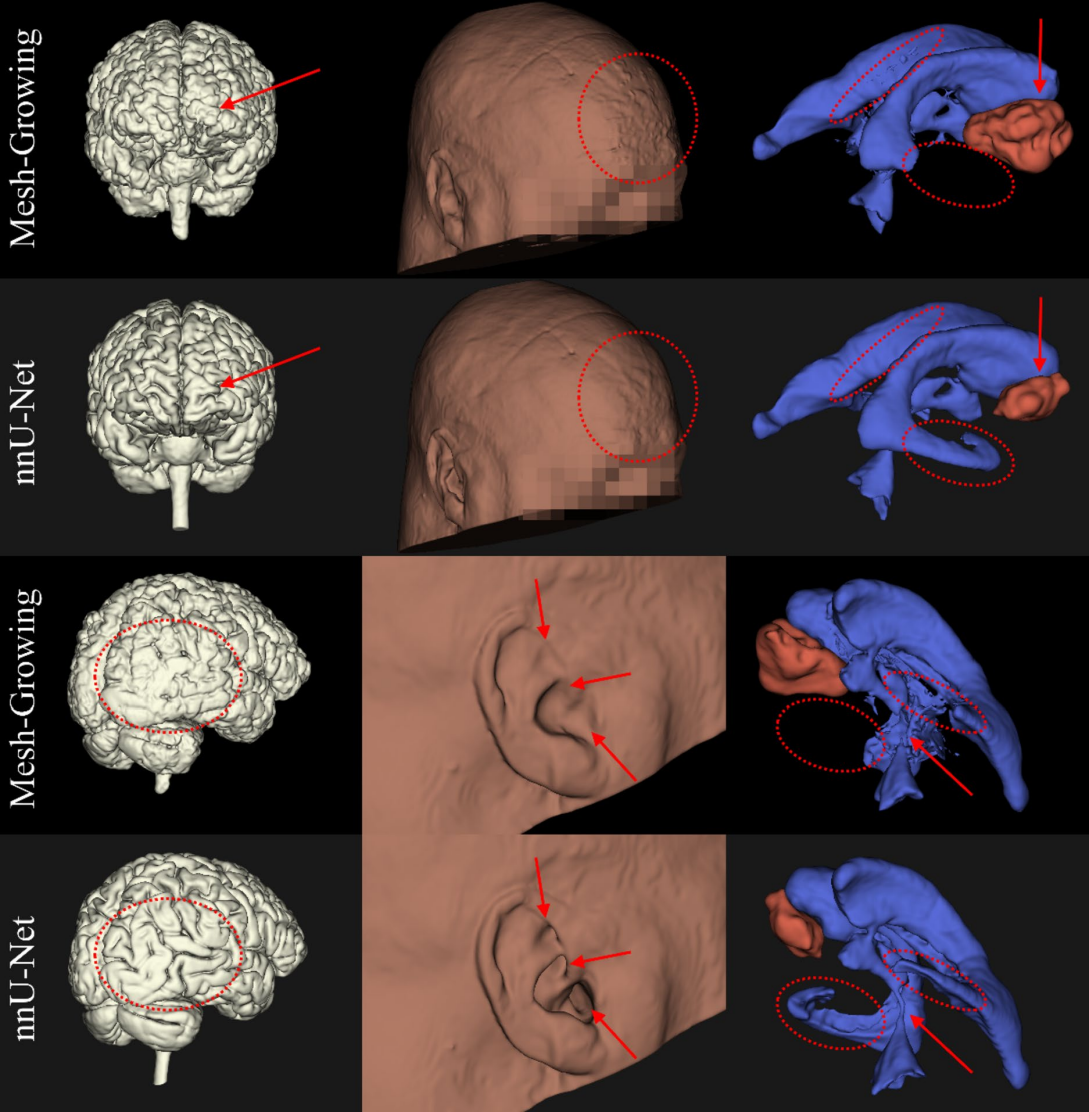
3D comparisons of segmentations generated by our nnU-Net models and the Mesh Growing Algorithm (MGA) (denoted row-wise in the margins). These visualize brain (left column, white), skin (middle column, brown), tumor (red, right column) and ventricles (blue, right column). The annotations in red indicate the same region in each segmentation with a notable difference in quality. The MGA oversegmented the tumor in this particular patient

**Fig. 2:**
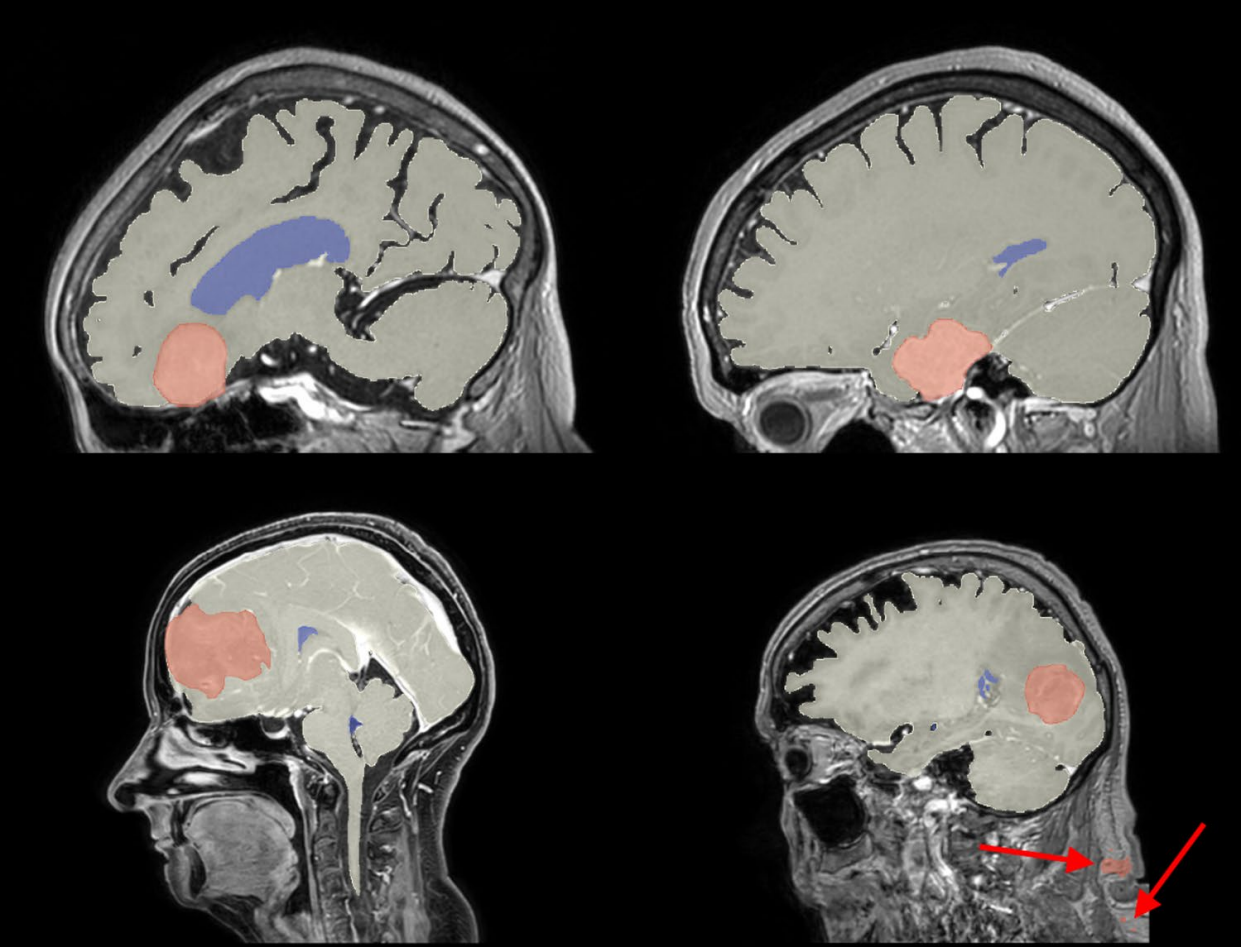
Example sagittal slices from our test set, indicating the difference in craniocaudal FOV, with the automatic brain (white), tumor (red) and ventricle (blue) segmentations overlaid. Each slice is positioned to display the bulk of the tumor. The top row are examples from Center A, the bottom row are examples from Center B. The red arrows are used to indicate false positives in the tumor segmentation

**Fig. 3:**
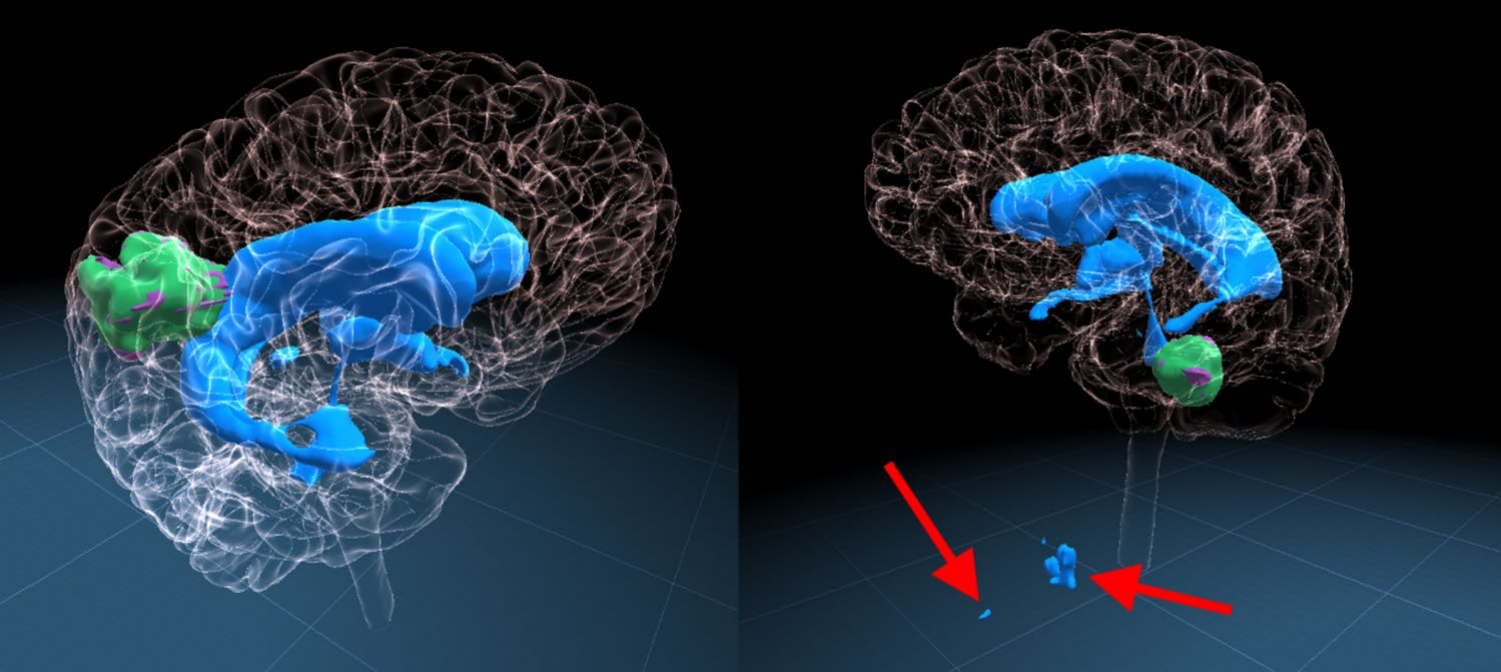
Typical example segmentations on two different patients (left: Center A, right: Center B) generated by our nnU-Net models. The brain segmentation is made transparent, to allow visualization of the underlying anatomy. the ventricle segmentations are blue, the tumor segmentation is green and the ground truth for the tumor is purple. Red arrows indicate false positive segmentations

The nnU-Net successfully produced all segmentations for each patient. The MGA failed to produce one brain, skin, and ventricle segmentation and five tumor segmentations. In our subsequent analyses, missing predictions were not included. Visually, the nnU-Net models produce a higher level of detail, reduced false positives and false negatives than the MGA, see Fig. 1 for examples. For a sagittal view of our nnU-Net model segmentations, including the T1CE scans they were derived from, see Fig. 2. Finally, since these models’ outputs are intended to serve as visualization aids, we have visualized them in an online environment, see Fig. 3.

**Fig. 4:**
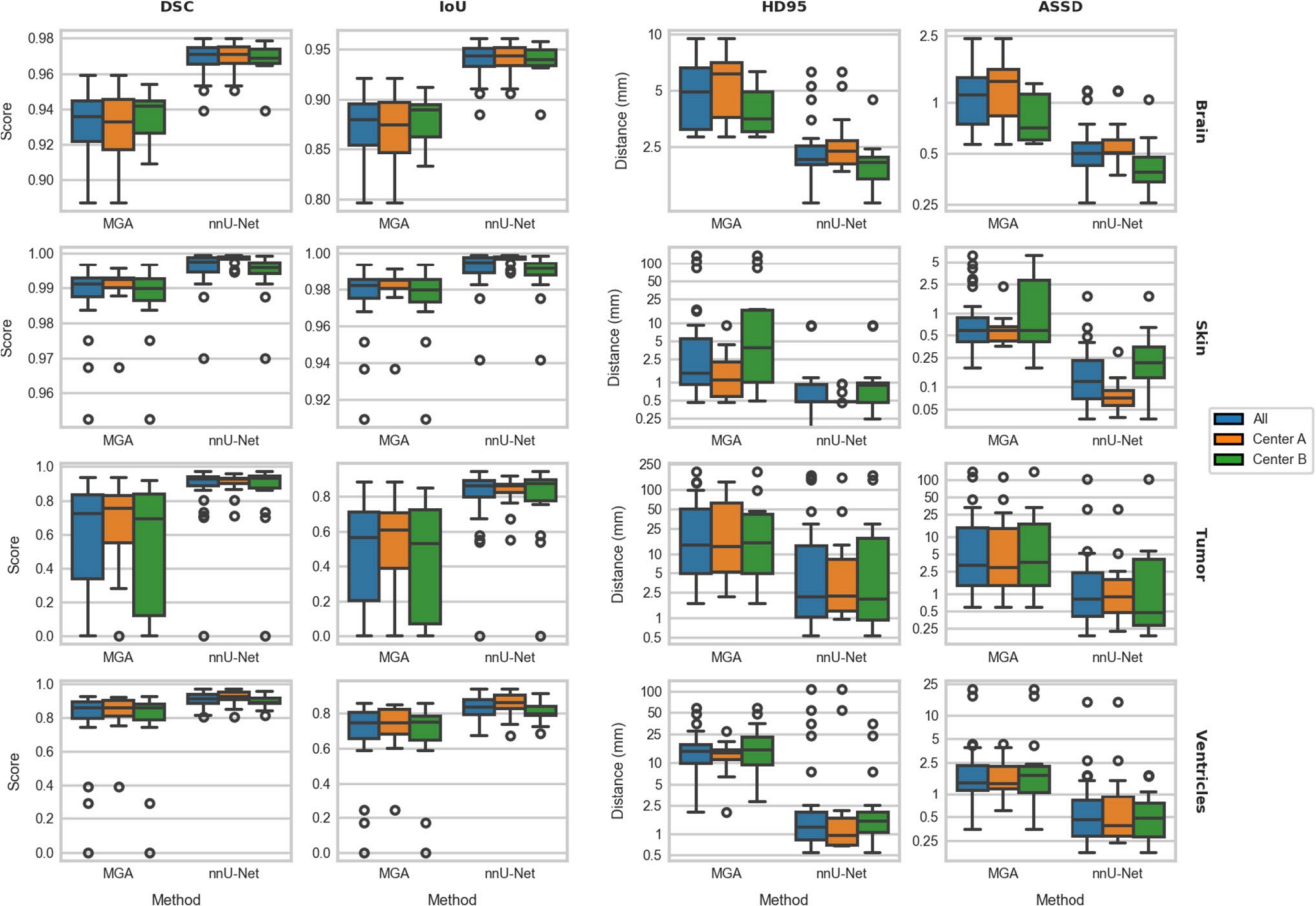
A boxplot showing performance of the MGA and our nnU-Net models side by side. The various anatomical structures are displayed in each row of plots, the used metrics are displayed in the columns. Note that the y-axes are independent to maximize visibility, and the HD95 and ASSD plots have a logarithmic y-axis

All nnU-Net models demonstrated superior performance for all different anatomical structures, with the largest difference observed in tumor segmentations. For a detailed breakdown of scores, readers are referred to Online Appendix A. A patient-specific comparison can be found in Online Appendix B. Figure 4 offers a visual representation of the achieved performance through boxplots. Finally, Appendix C contains an overfitting evaluation.

The results of the Mann–Whitney *U* tests, which compare the nnU-Net models and the MGA scores, suggest a statistically significant (*p* < 0.0125) difference in performance across all metrics and anatomical structures. Table 2 contains the results of these tests. A comparison of the inter-center performance of the nnU-Net models is available in Table 3. Apart from the skin segmentations, no statistically significant difference in performance was observed.

**Table 2:** Results of the Mann–Whitney U tests comparing our nnU-Net model segmentations versus those of the MGA. The tests used the alternative hypotheses that the nnU-Nets’ DSC and IoU were higher, and lower for HD95 and ASSD scores. U-values denoted by an asterisk have a *P*-value of < 0.001

Anatomy	DSC	IoU	HD95	ASSD
Brain	488*	488*	45.0*	47.0*
Skin	887*	887*	178*	77.0*
Tumor	649*	649*	184*	169*
Ventricles	793*	793*	124*	157*

**Table 3:** Results of the Mann–Whitney U tests comparing the performance of the nnU-Net models between both clinical centers A and B. U-values denoted with an asterisk have a *p-*value of < 0.001

Anatomy	DSC	IoU	HD95	ASSD
Brain	69.0 (*p* = 0.591)	69.0 (*p* = 0.591)	86.0 (*p* = 0.101)	94.0 (*p* = 0.0238)
Skin	224*	224*	83.0 (*p* = 0.0914)	34.0*
Tumor	97.0 (*p* = 0.534)	97.0 (*p* = 0.534)	120 (*p* = 0.787)	126 (*p* = 0.590)
Ventricles	177 (*p* = 0.0643)	177 (*p* = 0.0643)	85.0 (*p* = 0.113)	123.0 (*p* = 0.880)

On average, the nnU-Nets required 1139 s (19 min) [95CI: 685, 1616] to predict all anatomical structures sequentially. In contrast, the MGA took an average time of 2851 s (47.5 min) [95CI: 1482, 6246] to predict the same.

## Discussion

We propose nnU-Net as a promising option for generating automatic segmentation pipelines for brain, skin, contrast-enhancing tumor, and ventricles in T1CE scans. As MR systems continue to advance, on-demand, high-quality segmentations become increasingly vital. Beyond assisting experienced neurosurgeons, these segmentations can elevate the education of trainees, offering virtual rehearsal opportunities and a deeper grasp of complex anatomical relationships.

This study demonstrates that an nnU-Net trained on a relatively small dataset collected from a single center significantly outperforms the MGA. Except for skin segmentations, the nnU-Net is robust enough to not cause statistically significantly different performance between the two tested centers. With this, we provide future research endeavors the opportunity to redirect their energy towards using these segmentations for more advanced research that may provide a higher clinical impact, instead of focusing their efforts on solving the automatic segmentation problem instead.

Our models deliver predictions more rapidly than the MGA. While the MGA’s prediction times may be skewed due to unexpected background processes interfering or suboptimal hardware, our nnU-Net models remain substantially faster. Furthermore, although the nnU-Nets were timed sequentially, they can operate in parallel, given that sufficient computing power is available. Initial experimentation with running the models on more powerful hardware and combining all segmentations into a single model indicate the potential for a large amount of significant additional time savings.

### Prior works

Brain tumor segmentations are a popular subject, mainly due to the Brain Tumor Segmentation (BraTS) challenge [22] dataset. The existence of this open dataset lowers the barrier of entry for many researchers, resulting in a considerable amount of articles exploring the problem of automated brain tumor segmentation [1, 8]. However, nnU-Net surpassed the majority of these other works at the time of its publication [13], indicating its robust generalizability.

Despite potential benefits in using the BraTS data to train our own models, their data differs considerably from routine clinical data. Each set of scans in the BraTS dataset is extensively pre-processed with linear registrations, resampling to the same voxel spacing and skull stripping to preserve patient privacy. All of these steps require additional processing of the incoming data, which takes time and introduces additional points of failure in the pipeline. Our models do not require such pre-processing by design and are therefore more ergonomic in their integration in a clinical workflow.

DNN-based brain and ventricle segmentation methods have not been as widely explored as tumor segmentation, but several examples do exist. Most earlier works employ atlas registration techniques [4], which may take a lot of time to complete. In recent years, increasing amounts of DNN-based solutions were presented [2, 11, 36–38]. Generally, these studies focus on tissue classification or brain parcellation in the context of disease detection and/or disease progression assessments. In contrast, our segmentations aim to aid surgical planning, which focuses on the anatomical relations between the different brain structures, eliminating the need for such detailed differentiation.

While skin segmentation on its own is not a common subject of published research, it is a common step in analysis pipelines [15]. Often a thresholding technique is applied, which is subsequently processed with standard morphological operations [20]. While these techniques provide solid results without the need for training a model, each implementation is highly specific for the relevant dataset. In the presence of unexpected artefacts, signal loss and low-intensity regions these methods may fail to produce a proper mask.

Accurate and robust skin segmentations are crucial for preoperative planning. They provide valuable spatial information for locating the tumor relative to normally visible exterior reference points. Furthermore, this information could be used in the future to perform surface matching for neuronavigation. DNNs, like our nnU-Net models, can generate high-quality segmentations for these purposes.

### Limitations

We used a post-processing step to filter out false positive regions in our brain segmentations. This step was added to our process after initial results revealed a tendency of the model to generate these false positives in areas not seen in the training set. The scans from center B, which had a much larger craniocaudal field of view than center A, often included the neck. Since the scans from center A with an available manual brain segmentation did not include these areas, the model would have never been trained to recognize these areas. Fortunately, we know the brain segmentation should be one continuous structure. Removing all but the largest connected component improved the performance on these scans considerably.

However, this strategy does not translate well to tumor and ventricle segmentations. Removing all but the largest segmentations from these could introduce false negatives in the case of multiple tumors, slit ventricles, or other causes of component separation. It is not always known how many tumors any given patient has, nor is it straightforward to mathematically or programmatically reason which segmentations are false positives to be removed with a post-processing step. Furthermore, false negatives are difficult to detect. Post hoc user input may be required to indicate false positives or false negatives.

As is a common issue with DL solutions, not all segmentations may produce a correct result and/or these results may not generalize to a third clinical center. Fortunately, the high retrainability of a DNN allows future training runs to be performed on a dataset amended with data that has been the cause for low-quality segmentations. Prior research has indicated that only a small amount of additional out-of-distribution data may significantly improve performance on future iterations of the models [23].

Finally, we do not include failed segmentations in our statistical analyses. It could be argued that a failed segmentation should result in a DSC and IoU of 0.0, and a similar “complete miss” value for the HD95 and ASSD scores. Unfortunately, HD95 and ASSD do lack an upper bound [29] and should, in principle, be assigned a value of infinity. This would severely impact our statistical analyses to the point of unusability. As the MGA failed to produce several segmentations, while our nnU-Net models did not, our results are somewhat biased in favor of the MGA. Nevertheless, our nnU-Net models still outperform the MGA.

### Future applications

The models trained and tested in the current study accurately automatically segment skin, brain, tumors, and ventricles. This practically facilitates the creation of individual three-dimensional neurosurgical patient models from standard two-dimensional scans. The models are versatile in their display capabilities, suitable for presentation on conventional flat screens, advanced 3D displays, and augmented reality devices.

The potential implications of this technology in the context of neurosurgical patient care are multifaceted. The primary areas of impact include operative preparation, resident education, and patient education. The 3D models provide a platform for surgical planning and positioning rehearsals. They also serve as a foundation for creating immersive virtual simulations, enhancing the precision and preparedness of surgical interventions. These simulations are instrumental in the educational arena, particularly for training residents in fundamental surgical skills, including patient positioning and approach strategies. The 3D representations offer a more intuitive and detailed understanding of complex neuroanatomical structures. Finally, simplifying the complexity inherent in DICOM images, these 3D models offer a more comprehensible visual representation for patients. This approach can potentially improve patients’ understanding of their medical conditions, treatment plans, and the risks associated with various procedures.

Despite the apparent benefits, we underscore the necessity for further research to systematically assess and quantify the specific advantages and limitations of these 3D models in each of the aforementioned areas. This ongoing investigation is crucial for validating the efficacy and practicality of implementing such technology in clinical neurosurgical settings.

Future applications of the trained models in research are many. NnU-Net may empower researchers with a limited knowledge on DL pipelines to create their own powerful networks. This will allow them to focus on the actual use of the segmentations for clinically relevant research questions.

## Conclusion

We have demonstrated nnU-Net as an effective tool to develop automatic DL pipelines for segmenting brain, skin, contrast-enhancing tumors, and ventricles in T1CE scans. We also compared the performance of these trained models to a mathematical MGA, showing a statistically significantly improved performance on data from two neurosurgical hospitals.

## Supplementary Information

Below is the link to the electronic supplementary material.

Supplementary file1 (DOCX 18 kb)

Supplementary file2 (DOCX 16 kb)

Supplementary file3 (DOCX 159 kb)

## Data Availability

The training data includes patients’ faces, which cannot be freely distributed. The measurements will be made available alongside the code on https://github.com/MathijsdeBoer/nnUNet-vs-MGA.

## Code availability

The code for nnU-Net is available at https://github.com/MIC-DKFZ/nnUNet. The code for the statistical analyses and data handling will be made available at https://github.com/MathijsdeBoer/nnUNet-vs-MGA. Finally, when a suitable hosting solution is found, we will publish the trained models openly, so others may use them directly, or finetune them on their own dataset.
