# Supplementary material for: NnU-Net versus mesh growing algorithm as a tool for the robust and timely segmentation of neurosurgical 3D images in contrast-enhanced T1 MRI scans
Source: Acta Neurochir (Wien). 2024 Feb 20;166(1):92. doi: 10.1007/s00701-024-05973-8 (PMC10879314; doi:10.1007/s00701-024-05973-8)
Supplement: Supplementary file 1 — Supplementary file1 (DOCX 18 kb) [file 701_2024_5973_MOESM1_ESM.docx]

**Appendix A**

This table, split in two halves for clarity, shows the median scores and 95 confidence intervals (95CI) achieved by our nnU-Net models and the mesh-growing algorithm for each center and anatomy, as well as an overall mean score per anatomy. The values in brackets indicate the 95CI.

|  |  | **DSC** | | **IoU** | |
| --- | --- | --- | --- | --- | --- |
|  |  | nnU-Net  median, [95CI] | Mesh-Growing  median, [95CI] | nnU-Net  median, [95CI] | Mesh-Growing  median, [95CI] |
| Anatomy | Center |  |  |  |  |
| Brain | All | 0.971 [0.945, 0.979] | 0.936 [0.890, 0.958] | 0.943 [0.896, 0.959] | 0.879 [0.802, 0.920] |
|  | Center A | 0.971 [0.951, 0.979] | 0.933 [0.889, 0.959] | 0.943 [0.907, 0.960] | 0.874 [0.800, 0.920] |
|  | Center B | 0.969 [0.943, 0.978] | 0.941 [0.912, 0.953] | 0.939 [0.893, 0.956] | 0.889 [0.837, 0.910] |
| Skin | All | 0.997 [0.984, 0.999] | 0.991 [0.964, 0.996] | 0.995 [0.968, 0.999] | 0.982 [0.930, 0.992] |
|  | Center A | 0.999 [0.995, 0.999] | 0.992 [0.974, 0.995] | 0.997 [0.990, 0.999] | 0.985 [0.950, 0.990] |
|  | Center B | 0.996 [0.977, 0.999] | 0.990 [0.961, 0.996] | 0.991 [0.955, 0.997] | 0.980 [0.925, 0.992] |
| Tumor | All | 0.926 [0.508, 0.968] | 0.723 [0.000, 0.926] | 0.862 [0.391, 0.937] | 0.566 [0.000, 0.862] |
|  | Center A | 0.925 [0.744, 0.953] | 0.755 [0.0983, 0.917] | 0.86 [0.595, 0.91] | 0.607 [0.0571, 0.847] |
|  | Center B | 0.932 [0.245, 0.969] | 0.693 [9.93e-05, 0.916] | 0.873 [0.189, 0.94] | 0.531 [4.96e-05, 0.845] |
| Ventricles | All | 0.910 [0.812, 0.963] | 0.856 [0.216, 0.920] | 0.836 [0.683, 0.928] | 0.748 [0.127, 0.851] |
|  | Center A | 0.925 [0.819, 0.967] | 0.856 [0.518, 0.917] | 0.861 [0.695, 0.935] | 0.748 [0.369, 0.847] |
|  | Center B | 0.887 [0.825, 0.950] | 0.858 [0.108, 0.917] | 0.797 [0.702, 0.905] | 0.752 [0.0633, 0.847] |

|  |  | **HD95 (mm)** | | **ASSD (mm)** | |
| --- | --- | --- | --- | --- | --- |
|  |  | nnU-Net  median, [95CI] | Mesh-Growing  median, [95CI] | nnU-Net  median, [95CI] | Mesh-Growing  median, [95CI] |
| Anatomy | Center |  |  |  |  |
| Brain | All | 2.12 [1.45, 5.74] | 4.90 [2.81, 8.55] | 0.499 [0.301, 1.18] | 1.12 [0.569, 2.31] |
|  | Center A | 2.36 [1.84, 5.95] | 6.09 [2.82, 8.86] | 0.506 [0.407, 1.18] | 1.34 [0.627, 2.34] |
|  | Center B | 2.04 [1.31, 4.11] | 3.51 [2.83, 6.11] | 0.387 [0.269, 0.97] | 0.71 [0.575, 1.28] |
| Skin | All | 0.479 [0.189, 8.92] | 1.44 [0.477, 116] | 0.117 [0.0383, 0.882] | 0.583 [0.264, 5.1] |
|  | Center A | 0.479 [0.164, 0.958] | 1.11 [0.472, 7.41] | 0.0706 [0.0393, 0.243] | 0.583 [0.363, 1.81] |
|  | Center B | 0.898 [0.325, 9.04] | 3.87 [0.639, 127] | 0.214 [0.0572, 1.29] | 0.582 [0.223, 5.66] |
| Tumor | All | 2.11 [0.601, 158] | 13.6 [1.96, 154] | 0.825 [0.213, 49.4] | 3.23 [0.588, 121] |
|  | Center A | 2.16 [0.945, 116] | 13.0 [2.39, 130] | 0.888 [0.289, 21.3] | 2.95 [0.645, 87.6] |
|  | Center B | 1.95 [0.559, 160] | 14.9 [1.89, 169] | 0.477 [0.205, 67.3] | 3.62 [0.658, 113] |
| Ventricles | All | 1.24 [0.648, 66.2] | 14.4 [2.61, 50.7] | 0.466 [0.221, 5.34] | 1.38 [0.537, 18.7] |
|  | Center A | 0.938 [0.678, 89.2] | 13.7 [3.52, 24.9] | 0.389 [0.243, 10.4] | 1.35 [0.727, 4.15] |
|  | Center B | 1.48 [0.621, 30.9] | 15.3 [3.6, 54.7] | 0.487 [0.212, 1.72] | 1.71 [0.453, 20.2] |
