# Supplementary material for: NnU-Net versus mesh growing algorithm as a tool for the robust and timely segmentation of neurosurgical 3D images in contrast-enhanced T1 MRI scans
Source: Acta Neurochir (Wien). 2024 Feb 20;166(1):92. doi: 10.1007/s00701-024-05973-8 (PMC10879314; doi:10.1007/s00701-024-05973-8)
Supplement: Supplementary file 2 — Supplementary file2 (DOCX 16 kb) [file 701_2024_5973_MOESM2_ESM.docx]

**Appendix B**

This table indicates the mean difference with 95CI between the patient-wise scores achieved by our nnU-Net models and the mesh-growing algorithm. These were calculated by subtracting the metric value of the nnU-Net segmentation with the metric value of the mesh-growing algorithm for each patient where both methods had a successful segmentation. See the equation on this page. While failed segmentations could be marked with a DSC and IoU of 0.0, no such upper bound exists for HD95 and ASSD values^45^. This makes statistics calculations intractable for these cases. It is for that reason that we only consider successful segmentations.

$$D={Score}_{nnUNet}- {Score}_{MGA}$$

|  | | **DSC** | | **IoU** | |
| --- | --- | --- | --- | --- | --- |
| Anatomy | Center | Mean Diff, [95CI] | N | Mean Diff, [95CI] | N |
| Brain | All | 0.037 [0.015, 0.081] | 22 | 0.067 [0.027, 0.14] | 22 |
|  | Center A | 0.040 [0.019, 0.082] | 15 | 0.072 [0.036, 0.14] | 15 |
|  | Center B | 0.032 [0.014, 0.066] | 7 | 0.057 [0.025, 0.12] | 7 |
| Skin | All | 0.0073 [-1.4e-3, 0.020] | 31 | 0.014 [-0.00027, 0.037] | 31 |
|  | Center A | 0.0081 [0.0029, 0.022] | 15 | 0.016 [0.0057, 0.042] | 15 |
|  | Center B | 0.0065 [-0.0012, 0.016] | 16 | 0.013 [-0.0023, 0.029] | 16 |
| Tumor | All | 0.30 [-0.031, 0.92] | 25 | 0.34 [-0.043, 0.85] | 25 |
|  | Center A | 0.25 [-0.025, 0.82] | 15 | 0.29 [-0.030, 0.80] | 15 |
|  | Center B | 0.39 [-0.0062, 0.92] | 10 | 0.41 [-0.0086, 0.86] | 10 |
| Ventricles | All | 0.11 [-0.055, 0.61] | 31 | 0.14 [-0.088, 0.63] | 31 |
|  | Center A | 0.091 [-0.065, 0.40] | 15 | 0.13 [-0.099, 0.48] | 15 |
|  | Center B | 0.14 [-0.018, 0.75] | 16 | 0.16 [-0.030, 0.69] | 16 |

|  | | **HD95 (mm)** | | **ASSD (mm)** | |
| --- | --- | --- | --- | --- | --- |
| Anatomy | Center | Mean Diff, [95CI] | N | Mean Diff, [95CI] | N |
| Brain | All | -2.4 [-5.4, -0.075] | 22 | -0.59 [-1.5, -0.17] | 22 |
|  | Center A | -2.8 [-5.5, -0.012] | 15 | -0.69 [-1.6, -0.18] | 15 |
|  | Center B | -1.7 [-4.1, -0.37] | 7 | -0.37 [-0.81, -0.17] | 7 |
| Skin | All | -12. [-1.1e+02, 1.2] | 31 | -0.96 [-4.9, 0.079] | 31 |
|  | Center A | -1.4 [-6.9, 0.0] | 15 | -0.58 [-1.6, -0.25] | 15 |
|  | Center B | -23. [-1.3e+02, 2.2] | 16 | -1.3 [-5.5, 0.12] | 16 |
| Tumor | All | -21. [-1.3e+02, 36.] | 25 | -15. [-1.2e+02, 7.1] | 25 |
|  | Center A | -22. [-1.3e+02, 54.] | 15 | -13. [-86., 11.] | 15 |
|  | Center B | -20. [-73., 1.3] | 10 | -19. [-1.1e+02, -0.34] | 10 |
| Ventricles | All | -8.1 [-49., 57.] | 31 | -1.8 [-18., 4.8] | 31 |
|  | Center A | -1.8 [-24., 83.] | 15 | -0.26 [-3.9, 9.7] | 15 |
|  | Center B | -14. [-53., 12.] | 16 | -3.2 [-20., 0.89] | 16 |
