# Supplementary material for: NnU-Net versus mesh growing algorithm as a tool for the robust and timely segmentation of neurosurgical 3D images in contrast-enhanced T1 MRI scans
Source: Acta Neurochir (Wien). 2024 Feb 20;166(1):92. doi: 10.1007/s00701-024-05973-8 (PMC10879314; doi:10.1007/s00701-024-05973-8)
Supplement: Supplementary file 3 — Supplementary file3 (DOCX 159 kb) [file 701_2024_5973_MOESM3_ESM.docx]

**Appendix C**

We use this appendix to evaluate whether our models have been overfit on our training data. To assess this, we evaluate the performance of the model on the training data, and compare it to the performance on the test data. It is important to note that the performance on the training data is in no way representative of the actual performance of the model, and should only be regarded in the context of this comparative evaluation.


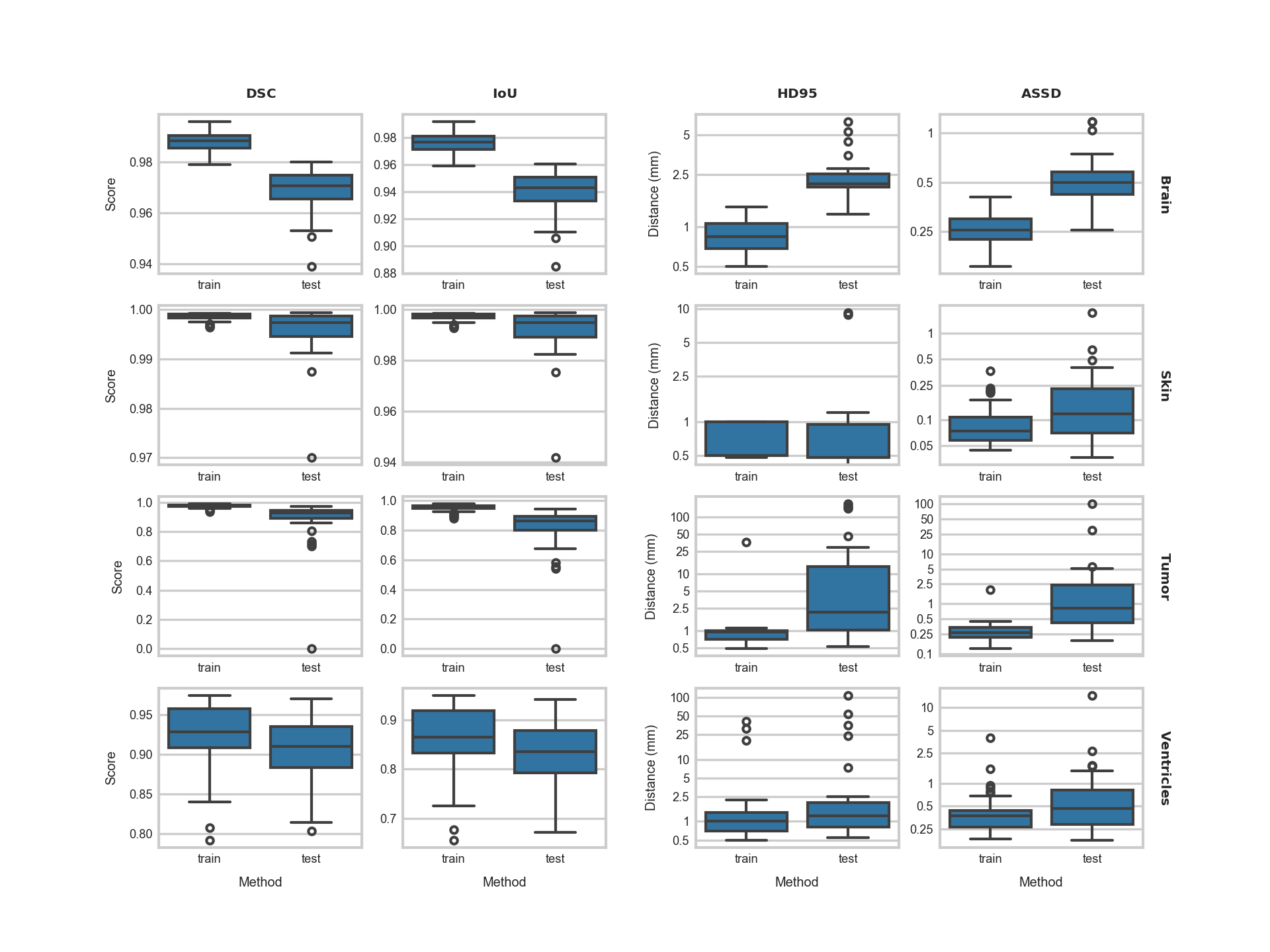


As is expected, the models perform better on the training data, as they have “seen” this information before, and have been specifically finetuned to minimize their error on this same data. However, as can be seen in the test performance, there are no large, unexpected differences in performance. We conclude that there are no signs of overfitting of our models based on this evaluation.
